# Risk Factors of Loneliness in Community-dwelling socially isolated older adults

**DOI:** 10.1093/geroni/igaf122.3119

**Published:** 2025-12-31

**Authors:** Eliza Lai-Yi Wong, Clement Cheuk-Wai Ng, Carol Ka-Po Wong, Shirley Shuk-KuenLui, Eng-Kiong Yeoh

**Affiliations:** The Chinese University of Hong Kong, Hong Kong, China (People’s Republic); The Chinese University of Hong Kong, Hong Kong, China (People’s Republic); The Chinese University of Hong Kong, Hong Kong, China (People’s Republic); The Chinese University of Hong Kong, Hong Kong, China (People’s Republic); The Chinese University of Hong Kong, Hong Kong, China (People’s Republic)

## Abstract

This study aims to identify the risk factors leading to loneliness in community-dwelling socially isolated older adults. The Lubben Social Network Scale-6 determined social isolation, whereas the UCLA 3-item loneliness scale assessed loneliness. A cross-sectional survey was conducted from September 2023 to February 2025, the age group and gender weightings were applied to ensure the population representative. Of the 596 older adults approached in the Hong Kong SAR, China, 269 (45.1%) were considered socially isolated, of which 62 (23.0%) socially isolated older adults experienced loneliness. Results of the logistic regression indicated that socially isolated older adults who attained primary education or below and had chronic disease had the odds ratio of 2.57 (95% CI: 1.31, 5.02) and 3.44 (95% CI: 1.15, 10.33) in undergoing loneliness comparing to those with secondary education or above, and healthy older adults respectively. Socially isolated older adults who reported deaf or severe hearing loss also revealed amplified loneliness risk with an odds ratio of 3.51 (95% CI: 1.04-11.90). Socially isolated older adults with poor quality of life of EQ-5D-5L utility ≤0.801 had enhanced loneliness risk with an odds ratio of 8.75 (95% CI: 3.05, 25.08) in contrast to those with EQ-5D-5L utility =1. Recognizing the leading factors to loneliness in socially isolated older adults provides insights to develop effective community interventions to combat loneliness and social isolation.

